# Increased axial resolution OCT improves structure-function correlation of the disorganization of the retinal inner layers in diabetic retinal disease

**DOI:** 10.1038/s41598-025-34931-2

**Published:** 2026-01-07

**Authors:** Katharina Wall, Lilith P. Arend, Leon von der Emde, Anna Sophia Jauch, Frank G. Holz, Marlene Saßmannshausen, Thomas Ach

**Affiliations:** Department of Ophthalmology, University Hospital Bonn, Venusberg-Campus 1, 53127 Bonn, Germany

**Keywords:** Diabetic retinal disease, Optical coherence tomography, OCT, disorganization of the retinal inner layers, DRIL, Structure-function correlation, Diseases, Endocrinology, Medical research

## Abstract

This study aimed to determine whether optical coherence tomography (OCT) with enhanced axial resolution improves structural characterization of the disorganization of the retinal inner layers (DRIL) in diabetic retinal disease (DRD) and its association with functional and vascular alterations. In this prospective cross-sectional study, 55 diabetic patients, 27 with DRIL (age: 57.1 ± 14.6 years) and 28 eyes without DRIL with diabetes mellitus (DM) type I/II (age: 58.2 ± 13.0 years), were examined with conventional spectral-domain OCT (SD-OCT), High-Resolution (High-Res) OCT, OCT angiography (OCTA), and microperimetry. DRIL areas were significantly smaller on High-Res OCT compared to SD-OCT (mean ± standard deviation, 0.3 ± 0.3 vs. 1.3 ± 1.2 mm^2^, *p* < 0.001). OCTA analyses demonstrated persistent group differences in superficial and deep vessel density and FAZ circularity between DRIL eyes and eyes without DRIL, even after adjustment for clinical diabetic retinopathy severity score (DRSS, all *p* < 0.05). Within DRIL eyes, intra-eye analysis revealed a stepwise reduction in retinal sensitivity from areas with DRIL detected on both OCT modalities (Zone I: 13.1 ± 1.3 dB) to areas with DRIL only on SD-OCT (Zone II: 20.6 ± 0.8 dB) and to unaffected retina (Zone III: 23.1 ± 0.7 dB, overall *p* < 0.001). OCT with enhanced axial resolution refines DRIL detection, aligning more precisely with localized functional and vascular impairment.

**Supplementary Information:**

The online version contains supplementary material available at 10.1038/s41598-025-34931-2.

## Introduction

Diabetic retinal disease (DRD) is a significant healthcare burden. In 2020, an estimated 103 million adults worldwide were affected by DRD, and the incidence is expected to increase^1^. The pathogenesis of DRD is intricately linked to the dysfunction of the neurovascular unit (NVU), which is characterized by neuroinflammation, neurodegeneration and microvascular changes^2–8^.

In clinical studies, conventional spectral-domain optical coherence tomography (SD-OCT) biomarkers in DRD^9–13^ have been demonstrated as predictors for both functional and anatomical outcomes. Among these, the disorganization of the retinal inner layers (DRIL) has emerged as a significant indicator for neural retinal health^14,15^. In 2014, Sun et al. defined DRIL as the extent of microns within the central 1000 μm for which the boundaries between the ganglion cell layer (GCL), the inner plexiform layer (IPL), the inner nuclear layer (INL), and the outer plexiform layer (OPL) could not be identified^16,17^. DRIL is negatively correlated with best corrected visual acuity (BCVA)^16–19^ probably caused by a dysfunctional communication and later apoptosis^20^ of photoreceptors and ganglion cells^19,21^ as well as a major dysfunction of Müller cells^20,22^. In addition, DRIL is associated with microvascular changes in all retinal capillary plexus^23^ and with an enlargement of the foveal avascular zone (FAZ)^24^, clinically detectable on OCT angiography (OCTA)^23–26^.

While BCVA is a key outcome in clinical studies, it may not capture all functional impairment in DRD, particularly in early stages when BCVA is unaffected^27^. Retinal damage resulting from DRD leads to a reduction in contrast sensitivity and impaired color vision even before impairment in BCVA^27–30^. Regulatory agencies increasingly prioritize functional outcomes^31^, e.g. fundus controlled perimetry, to assess the topographic functional impact of structural alterations^27,32^. Previous studies have demonstrated that the mean retinal sensitivity is impaired in patients with diabetic macular ischemia^25,33^ and DRIL^34^.

Recent advancements in OCT technology have led to the development of systems with markedly improved axial resolution, while lateral resolution remains unchanged. The High-Resolution (High-Res) OCT used in this study achieves an axial resolution of 2.9 μm compared to 7.2 μm in the previous conventional spectral-domain OCT (SD-OCT) device by the same manufacturer. This improvement is accomplished by employing a shorter central wavelength (853 nm instead of 880 nm) and increased spectral bandwidth (from 50 nm to 137 nm)^35,36^. These improvements increased inner retinal band visibility^36^ and enable more precise delineation of microstructural abnormalities in DRD^37^ and age-related macular degeneration (AMD)^36,38^.

We recently showed significantly smaller DRIL areas in improved axial resolution (High-Res OCT) compared to conventional SD-OCT; however, the functional relevance of this finding is yet to be understood^37^. We hypothesized that DRIL defined by High-Res OCT corresponds more closely with localized retinal ischemia and functional impairment, due to an improved visualization of structural pathologies. The aim of this study was to evaluate whether OCT with enhanced axial resolution improves the correspondence between DRIL and associated functional and microvascular impairment in DRD, compared to conventional SD-OCT.

## Results

### Cohort characteristics

A total of 55 diabetic patients (55 eyes, 27 with DRIL; 28 eyes without DRIL) were enrolled. Cohort characteristics are plotted in Table 1. Groups differed significantly in BCVA (DRIL-group: 0.35 ± 0.32 vs. no DRIL group: 0.09 ± 0.1 logMAR, *p* < 0.001), duration of diabetes (19.71 ± 14.62 vs. 9.22 ± 6.98 years, *p* = 0.03) and DRSS (4.81 ± 0.47 vs. 1.21 ± 0.41, *p* < 0.001). Most patients in the DRIL group were insulin-dependent (55.6%), whereas oral therapy predominated in the no DRIL group (32.1%). Cardiovascular comorbidities were frequent in both groups (85.2% vs. 75.0%, *p* = 0.22).

### Structural imaging

En-face DRIL extensions in High-Res OCT (30° × 25°, ART 25, 121 B-scans, High-Resolution mode; axial resolution: 2.9 μm, Heidelberg Engineering, Heidelberg, Germany) were significantly smaller than in SD-OCT (30° × 25°, ART 25, 121 B-scans, High-Resolution mode, Spectralis HRA-OCT 2; Heidelberg Engineering, Heidelberg, Germany) (0.31 ± 0.29 mm^2^ vs. 1.26 ± 1.24 mm^2^, *p* < 0.001), confirming our previous findings^57^. DRIL was identifiable on both SD-OCT and High-Res OCT in all included eyes, and no cases of DRIL detectable exclusively on one device were observed. Intraclass correlation coefficient (ICC) for DRIL areas on SD-OCT was 0.77 and for High-Res OCT 0.82.

**Fig 1:**
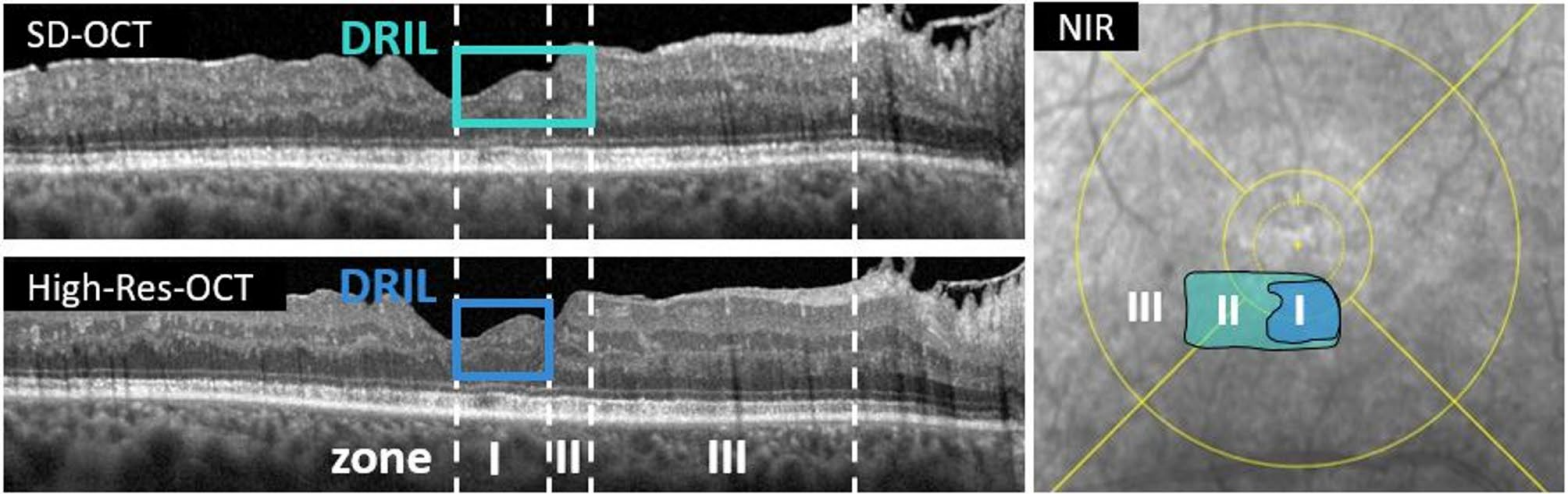
Macular B-scan of SD- and High-Res OCT and near-infrared reflectance (NIR) image of a 49-year-old patient with DRIL. DRIL is defined as the loss of discernibility of the inner retinal layers, including RNFL, GCL, IPL, INL, and OPL. Compared to SD-OCT, the High-Res OCT allows for clearer layer differentiation and reveals significantly smaller DRIL areas. This difference is visualized in the NIR image with en face overlays: DRIL areas detected on SD-OCT are marked in turquoise, while those detected on High-Res OCT are shown in blue. Based on these findings, three zones were defined: (I) DRIL detectable on both OCT modalities, (II) DRIL detectable on SD-OCT but not on High-Res OCT, and (III) no DRIL detectable on either modality.

**Fig 2:**
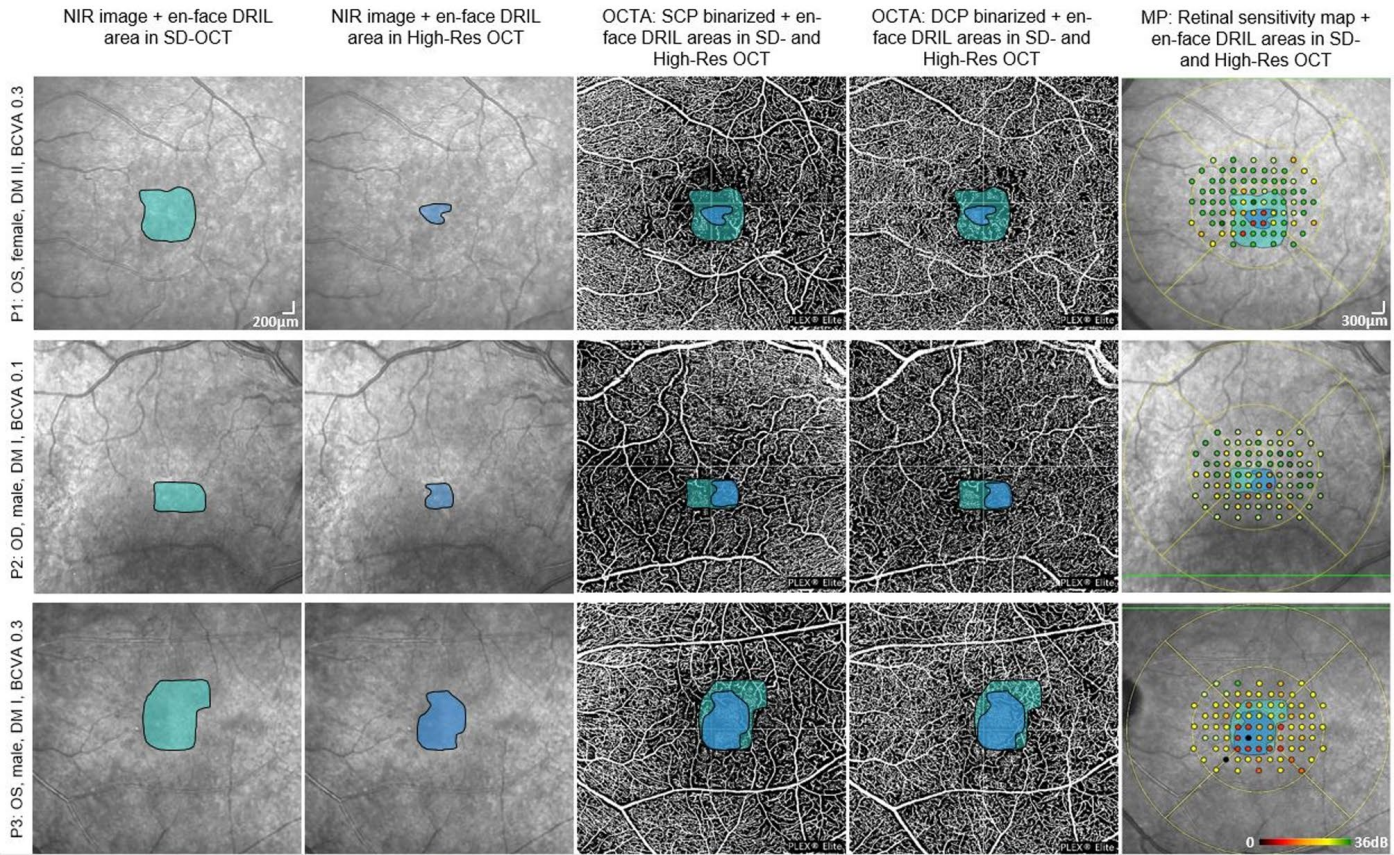
Superimposition of en face DRIL extensions (SD-OCT and High-Res OCT) with binarized SCP and DCP data as well as retinal sensitivity maps in three representative patients (P1-P3). En face DRIL maps were generated from segmentation of inner retinal layers on SD-OCT (turquoise) and High-Res OCT (blue). Overlays were co-registered with the NIR image, binarized OCTA of SCP and DCP, and mesopic microperimetry maps. Microperimetry sensitivity is color-coded: green/yellow (≥ 20 dB), orange (10–20 dB), and red (< 10 dB). There is a clear loss in retinal sensitivity in DRIL areas detected on High-Res OCT. NIR: Near-infrared reflectance; SCP: Superficial capillary plexus; DCP: Deep capillary plexus; MP: Microperimetry; OS: Oculus sinister; OD: Oculus dexter; DM: Diabetes mellitus.

Zone-based (Figure 1) OCTA (Swept-Source OCT-Angiography, scans of 6 × 6 mm (500 horizontal A-scans, 6.3 μm axial resolution)) analyses in DRIL eyes demonstrated a spatial gradient of vascular impairment (Table 2; Figure 2). In the deep vascular plexus (DCP), vessel density (VD) declined from 9.60 ± 0.35 in Zone III to 7.21 ± 0.31 in Zone I, with significant pairwise differences between Zone I and Zone III (*p* < 0.001) and Zone II and Zone III (*p* < 0.001). DCP perfusion density (PD) showed a similar gradient (33.04 ± 1.65 in Zone III vs. 21.16 ± 1.52 in Zone I, *p* < 0.001). In the superficial capillary plexus (SCP), PD was also lower in DRIL-affected zones (18.45 ± 1.49 in Zone I vs. 32.59 ± 1.48 in Zone III, *p* < 0.001), although overall differences across all zones did not reach significance.

At the group level (Table 3), analyses revealed significant reductions in both SCP (VD 7.36 ± 0.81 vs. 11.61 ± 0.63, *p* = 0.004; perfusion density 28.88 ± 3.20 vs. 42.92 ± 2.48, *p* = 0.02) and DCP (vessel density 6.93 ± 1.08 vs. 12.48 ± 0.08, *p* = 0.01; PD 25.01 ± 5.26 vs. 44.15 ± 4.09, *p* = 0.04) in DRIL eyes compared to eyes without DRIL, adjusted for DRSS and diabetes duration. FAZ circularity was also significantly reduced in DRIL eyes (SCP: 0.56 ± 0.09 vs. 1.01 ± 0.07, *p* = 0.01; DCP: 0.55 ± 0.10 vs. 0.96 ± 0.08, *p* = 0.02). Unadjusted results, which showed broader differences in FAZ size and perimeter, are provided in Table S1.

### Functional testing

In total, 4001 microperimetry (MP, CenterVue/iCare, Oy, Finland) stimuli points were analyzed: 2116 from DRIL eyes and 1985 from eyes without DRIL. Within the DRIL group, 100 points were located in Zone I, 46 points in Zone II, and 1045 points in Zone III. The remaining 925 points showed a combination of DRIL with ME/DROL or ME/DROL without DRIL and were therefore excluded from the intra-eye zone analysis. The inter-reader reliability for microperimetry grading was κ = 0.70.

In DRIL eyes, RS showed a strong zonal gradient (Table 2): Zone I had the lowest values (13.07 ± 1.28 dB), followed by Zone II (20.62 ± 0.79 dB, *p* = 0.016 vs. Zone I) and Zone III (23.11 ± 0.73 dB, *p* = 0.002 vs. Zone I; *p* = 0.020 vs. Zone II).

Across retinal locations (Table 4), adjusted models confirmed that RS was significantly reduced in DRIL areas (17.6 ± 1.83 dB) compared with non-pathological retina in the same eyes (27.6 ± 1.78 dB, *p* < 0.001). RS deviation analyses mirrored this pattern, with the largest functional loss localized to DRIL areas (– 8.9 ± 1.83 vs. non-pathological retina, *p* < 0.001). Full adjusted values are reported in Table 4 and unadjusted values are shown in Table S2.

### Structure-function relationship

Correlation analyses confirmed strong associations between structural and functional outcomes (Table S3). Lower BCVA correlated with reduced SCP and DCP vessel and skeleton density (all r between − 0.39 and − 0.44, *p* ≤ 0.02), larger FAZ size (*r* = 0.40, *p* = 0.01) and perimeter (*r* = 0.48, *p* < 0.001), and reduced FAZ circularity (*r* = − 0.39, *p* = 0.018). RS also correlated strongly with BCVA (*r* = − 0.50, *p* < 0.001).

### Post-hoc power analysis

A post hoc power analysis was performed to assess the statistical sensitivity of the study after adjustment for DRSS and diabetes duration. For the microperimetry-based comparison of RS across DRIL-defined zones (Zone I–III), the ANCOVA yielded a sufficient effect size (Cohen’s *f* = 1.09) resulting in an estimated statistical power of > 99.9% (*α* = 0.05). For OCTA parameters, comparisons were based on the patient-level sample (*n* = 55). Sufficient effect sizes were observed for both SCP (Cohen’s *f* = 0.94) and DCP (Cohen’s *f* = 0.83), with power again exceeding 99.9% in both cases.

## Discussion

This study evaluated whether differences in the extent of DRIL areas delineated by conventional SD-OCT and improved axial resolution High-Res OCT can be linked to local macular perfusion and retinal sensitivity. The principal finding was a clear intra-eye gradient of vascular parameters and retinal sensitivity across predefined zones: the most pronounced deficits were observed in regions identified as DRIL by both SD- and High-Res OCT (Zone I), intermediate impairment occurred in SD-OCT–only DRIL regions (Zone II), and function was relatively preserved in unaffected retina (Zone III). These results demonstrate that High-Res OCT provides a more restricted but functionally more relevant delineation of DRIL, while additional areas detected on SD-OCT are not artefactual but may represent earlier or milder stages of structural disruption.

### Microvascular alterations associated with DRIL

OCTA revealed significant vascular alterations in DRIL eyes. After adjusting for DRSS and diabetes duration, both SCP and DCP vessel and perfusion densities remained significantly reduced compared to eyes without DRIL, and FAZ circularity was impaired in both plexuses. These findings establish reduced VD and PD as well as altered FAZ geometry as robust vascular correlates of DRIL, independent of systemic disease burden. Correlation analyses confirmed that lower VD and SD, larger FAZ size and perimeter, and reduced circularity were linked to worse BCVA, highlighting the functional relevance of these microvascular abnormalities, coherent with previous studies^39–43^.

Intra-eye zonal analysis demonstrated a stepwise gradient of vascular impairment: VD and PD were lowest in Zone I, intermediate in Zone II, and highest in Zone III. The gradient was most pronounced in the DCP, consistent with previous reports that capillary dropout in the deep plexus typically precedes changes in the SCP^44–47^. OCTA metrics have been widely studied in diabetic patients, because of their possibility to evaluate perfusion status, disease severity and prognosis without invasive imaging^40,48–52^. Previous OCTA studies have consistently shown that DRIL regions correspond to areas of capillary non-perfusion^39,53–55^, particularly involving the DCP and, in some cases, the SCP or intermediate capillary plexus^23,34,56^. FAZ enlargement, lower VD, and reduced FAZ circularity have been identified as sensitive indicators of macular ischemia and functional loss^34,39,56,57^.

Our data extends previous observations by demonstrating that vascular impairment is closely aligned with DRIL as defined by High-Res OCT, supporting DRIL as a structural marker of macular ischemia and highlighting that improved resolution strengthens the link between vascular pathology and functional impairment.

### Microperimetry findings

We hypothesized that High-Res OCT would identify DRIL regions that align more precisely with localized functional impairment measured by pointwise fundus-controlled microperimetry. Although the original definition of DRIL is confined to an area of 1000 μm centered at the fovea^16^, it was shown that DRIL extends more widely across the macula. Restricting the evaluation to the central 1000 μm may therefore underestimate its overall extend and functional impact. By assessing DRIL within the central 3 mm, our study enabled a more comprehensive characterization of its topographic distribution and a precise pointwise structure-function correlation.

Intra-eye analyses confirmed that the stepwise gradient observed in vascular parameters was mirrored by retinal sensitivity: sensitivity was lowest in Zone I, intermediate in Zone II, and highest in Zone III. This underscores the clinical benefit of High-Res OCT for improved visualization of DRIL, which has a direct impact on retinal function.

Between-group analyses revealed an additional layer of complexity. DRIL eyes showed markedly lower retinal sensitivity compared to eyes without DRIL in unadjusted models, but after adjustment for DRSS and diabetes duration, the overall difference was no longer significant. This suggests that systemic disease severity and chronicity account for much of the group-level disparity. However, within DRIL eyes, sensitivity remained nearly 10 dB lower in DRIL affected areas compared to adjacent non-DRIL areas even after adjustment. Thus, DRIL itself remains a strong local marker of focal dysfunction, independent of systemic disease burden.

Our results align with and extend prior reports on the functional implications of DRIL. Vujosevic et al.^34^ demonstrated a significant reduction in mean foveal retinal sensitivity (central 1 mm) in patients with DRIL compared to non-DRIL subjects (*p* < 0.001). Alonso-Plasencia et al.^33^ showed that sensitivity loss co-localizes with microvascular abnormalities temporal to the fovea, while Tsai et al.^32^ further reported that sensitivity below 25 dB differentiated eyes with structural abnormalities, including DRIL, FAZ enlargement, and reduced vessel density, from less severe cases. Consistent with these observations, even non-pathological areas within DRIL eyes in our study exhibited mean sensitivity below 25 dB, whereas eyes without DRIL remained at this threshold, as seen in the unadjusted RS values (Table S2). In summary, sensitivity loss was most pronounced in regions where DRIL was identified by both SD- and High-Res OCT, while areas detected only by SD-OCT showed a milder degree of impairment. This gradient suggests that High-Res OCT delineates DRIL areas with the strongest functional relevance, whereas SD-OCT may additionally capture earlier or less advanced stages of inner retinal disorganization.

### Influence of additional biomarkers, limitations, and strengths

The potential impact of other structural biomarkers cannot be fully excluded, particularly if such alterations were transient or had resolved prior to imaging, as they may still influence retinal sensitivity. In particular, macular edema has been closely linked to DRIL, and even after resolution, residual structural alterations may contribute to sensitivity loss. In addition, outer retinal changes such as disruption of the ellipsoid zone (EZ) or external limiting membrane (ELM) may also affect retinal sensitivity. In our intra-eye analyses, 925 microperimetry stimulus points were excluded because of coexisting EZ/ELM alterations or macular edema, underlining the frequency with which these biomarkers overlap with DRIL. While this approach ensured that the observed functional deficits could be attributed more specifically to DRIL, it also illustrates the challenge of disentangling DRIL from other retinal abnormalities in DRD. Notably, no cases with extensive macular edema were included, as reflected by comparable central subfield thickness (CST) between DRIL group and no DRIL group. Future longitudinal studies should address the combined and potentially interacting influence of DRIL, EZ/ELM integrity, and macular edema dynamics on visual function.

Other limitations include the modest sample size (*n* = 55), single center design, and the cross-sectional nature, which preclude conclusions about temporal progression. The relatively high exclusion rate of DRIL patients (*n* = 21, Figure S1) was mainly due to suboptimal image quality (as defined in the methods) and fixation instability in eyes with advanced DRIL. As DRIL is prone to intergrader variability and precise multimodal alignment requires high-quality data, only eyes with reliable registration and fixation-stable microperimetry testing were included. Additionally, we now note that this selection likely resulted in the exclusion of patients with very advanced structural alterations, as these eyes often cannot perform all imaging and functional tests with sufficient quality. Consequently, our study population represents eyes with mild-to-moderate DRIL severity. Comparisons of retinal sensitivity between DRIL and no DRIL eyes were based on averaged values within predefined zones rather than strictly matched stimulus locations. Retinal sensitivity deviation analysis partly addressed this by referencing normative pointwise control values, but residual regional variability^58,59^ cannot be fully excluded. Minor misalignments between SD- and High-Res OCT may have occurred due to the lack of follow-up mode, and the uniform size of microperimetry stimuli (~ 120 μm), which restricted resolution for small lesions. Lesion-specific grids may improve precision in future studies.

Strengths of this study include its methodological approach of directly comparing DRIL delineation between conventional SD-OCT and High-Res OCT. This head-to-head evaluation enabled us to test whether the higher axial resolution of High-Res OCT improves the specificity of structure–function correlations. By aligning en-face DRIL maps with both OCTA and pointwise microperimetry, we demonstrated concordant gradients of vascular and functional impairment across zones, reinforcing DRIL as a biomarker of localized ischemia. While multimodal imaging in DRD has been reported previously, our study adds the novel dimension of cross-device comparison, showing that High-Res OCT reduces overestimation of pathological areas and enhances the precision of DRIL characterization.

## Conclusion

DRIL is associated with local sensitivity loss and microvascular alterations in DRD. High-resolution OCT identifies smaller but functionally more significant DRIL-affected areas than conventional SD-OCT, thereby improving the accuracy of structure-function correlations. While systemic disease burden explains part of the group-level differences between DRIL and eyes without DRIL, DRIL itself remains a robust local marker of focal dysfunction and ischemia. Future longitudinal studies are warranted to determine whether High-Res OCT–based DRIL assessment improves prediction of visual outcomes and disease progression, and how coexisting biomarkers such as EZ/ELM alterations and macular edema influence its prognostic value.

## Methods

### Patient selection

This prospective cross-sectional study was conducted at the University Eye Hospital Bonn, Germany, between July 2023 and February 2025. The ethics committee of the University of Bonn approved the study protocol (#301/21) and this research adhered to the Tenets of the Declaration of Helsinki. Before participating, each patient provided written informed consent. Patients aged 18 years or older with type 1 or type 2 diabetes mellitus (DM) and DRIL within 3 mm centered on the fovea and detectable on both OCT devices (SD-OCT, High-Res OCT) were enrolled. Exclusion criteria were the presence or history of any other retinal disease, including AMD, retinal dystrophy, pathologies of the vitreomacular interface, full thickness macular or lamellar macular holes, retinal vein or artery occlusion, or poor image quality due to optic media opacities (e.g., dense cataracts, vitreous floaters) and refraction errors (> 6 Diopters spherical, > 2 Diopters cylindrical). There were no cases where both eyes met the inclusion criteria.

In total, 48 patients with DRIL were initially screened; however, only 27 met all inclusion criteria and showed sufficient image quality in both OCTA and MP and were therefore included in the final analysis (further mentioned in the manuscript as “DRIL group”). 21 screened patients were excluded primarily due to fixation instability, which led to motion artifacts in OCTA imaging and high variability in microperimetry testing. Age-matched DM patients with no or only mild DRD (diabetic retinopathy severity scale 1 or 2)^60^ and without DRIL served as controls. Twenty-nine age-matched controls were screened and 28 included in the final analysis (further mentioned as “no DRIL group” or “eyes without DRIL”, Fig. 1). All eyes, both with and without DRIL, were examined using identical multimodal imaging protocols and evaluated according to the same grading criteria.

### Systemic risk factors and disease severity assessment

Patient self-reporting by an interview was used to collect medical information, which included the specific type of diabetes, duration since initial diabetes diagnosis, current HbA1c level, current diabetes therapy and cardiovascular risk factors including arterial hypertension, hypercholesterinemia, previous cardiovascular event (e.g., myocardial infarction or stroke), smoking, and/or obesity according to the definition of the World Health Organization definition^61^. To assess the severity of DRD, we employed the international clinical diabetic retinopathy severity scale (DRSS)^60^ based on a dilated fundus examination performed by a clinician (K.W.).

### Image acquisition

All subjects underwent BCVA testing, slit lamp biomicroscopy of the anterior segment, and indirect funduscopy (after pupil dilation with 1.0% tropicamide and 2.5% phenylephrine) prior to retinal imaging. The retinal imaging protocol included conventional SD-OCT (30° × 25°, ART 25, 121 B-scans, High-Resolution mode, Spectralis HRA-OCT 2; Heidelberg Engineering, Heidelberg, Germany), High-Res-OCT (30° × 25°, ART 25, 121 B-scans, High-Resolution mode; axial resolution: 2.9 μm, Heidelberg Engineering, Heidelberg, Germany), OCTA (Swept-Source OCT-Angiography, scans of 6 × 6 mm (500 horizontal A-scans, 6.3 μm axial resolution), Zeiss PLEX Elite 9000, Carl Zeiss Meditec, Dublin, California, USA) and color fundus photography (131° Clarus, Carl Zeiss Meditec AG, Jena, Germany).

### Retinal sensitivity assessment

The S-MAIA device (CenterVue/iCare, Oy, Finland) was used for mesopic retinal sensitivity examination. The test configuration included 85 stimuli within the central 12° of the retina covering the central and inner ETDRS ring, utilizing a 4 − 2 dB staircase strategy with a Goldmann III stimulus size of 0.43°, a background luminance of 4 apostilb (1.3 cd/m2) and a 36 dB dynamic testing range^62^. Fixation tracking speed was set at 25 Hz. Patients underwent a preliminary training session before the main examination. The final microperimetry results where projected automatically on a near-infrared reflectance (NIR) image, simultaneously captured during testing^63^. For each stimulus, besides the retinal sensitivity (RS) values, pointwise RS deviation was calculated as the difference between the measured sensitivity in DRIL eyes and the normative mean of eyes without DRIL at the same location.

### OCT analysis

Volumetric SD-OCT and High-Res OCT imaging data were automatically segmented using the device’s internal software (Spectralis Viewer Module 6.3.2.0; Heidelberg Engineering Eye Explorer, Heidelberg, Germany), reviewed in each of the 121 B-scans and manually corrected, if necessary, based on IN-consensus^64^. Each retinal layer in each cross-section OCT scan was assessed for its visibility and ability to be separated from the neighboring layer. The loss of discernibility was manually annotated in retinal scan using the device´s internal software. In our study, DRIL was defined as a discernibility loss of all retinal inner layers (RNFL, GCL, IPL, INL, OPL). DRIL extensions in the cross-section OCT scans were then converted into an en-face image and further processed in Image J^65,66^. The en-face DRIL areas were binarized, converted to mm^2^ and an ETDRS grid was projected, centered at the fovea. This entire procedure was performed identically for both OCT modalities.

### OCTA analysis

OCTA images were first screened by a trained grader (K.W.) for image quality, and images showing significant artifacts, e.g., motion artifacts, or reduced OCTA signal (minimum 7/10 graded from the internal software of the Plex Elite 9000) were excluded. OCTA images were then semiautomatically segmented (ILM to OPL) and manually adjusted if necessary. After segmentation, OCTA images were exported and further processed in Image J. The size and the perimeter of the FAZ were manually measured and FAZ circularity was calculated using the following formula^67^: 

$\:Circularity\:=\:4\pi\:\:x\:FAZ\:area\:/\:\left(FAZ\:perimeter\right)$^2^. Then, we used the ‘Otsu’ thresholding method for binarization of the SCP and DCP images^68^. Afterwards, the images were skeletonized and the ´Analyze particles´ function was used for assessing vessel density (VD) and perfusion density (PD) for the whole image^69^. OCTA images were then aligned with the en-face DRIL areas using the Image J Plugin “Manual landmark selection” using vessel bifurcations in three to four quadrants of the image as markers. Afterwards VD/ PD of the SCP and the DCP were measured individually for zone I, II, and III. The zone masks (Zones I–III) were imported into the OCTA analysis workspace and applied as binary overlays, ensuring that VD and PD were quantified within identical spatial regions across modalities.

### Multimodal image alignment

The microperimetry sensitivity maps were co-registered with the en-face DRIL maps in Image J (Plugin: “Registration MP OCT”) using retinal vascular bifurcations and distinct vessel crossings (marks in three to four quadrants of the image) as fiducial markers, to ensure precise spatial correspondence between the different imaging modalities^65^. For each eye, the near-infrared fundus image obtained during MP acquisition was aligned manually to the en-face OCT projection using rigid registration (translation and rotation only). Scaling adjustments were automatically performed by the Plugin. The alignment accuracy was visually confirmed by both graders (K.W. and L.P.A.) through overlay inspection at high magnification. In cases with misalignment the process was repeated.

After the alignment of all imaging modalities, each MP stimulus was graded by two readers (K.W. and L.P.A.) for the following categories:

1. DRIL (yes/no).
2. Macular edema (ME) (yes/no).
3. Disorganization of the outer retinal layers (DROL), defined as reflectivity loss of the ELM and/or EZ^70^.

This grading was performed seperately for both OCT modalities and their respective DRIL maps.

Based on our previous study, we defined three OCT-based zones: (I) DRIL detectable on both OCT devices (abbr. High-Res DRIL), (II) DRIL detectable in SD-OCT, but not High-Res OCT (abbr. SD-/High-Res DRIL), and (III) no visible pathologies on both OCT devices (abbr. no DRIL). (Fig. 1). For the intra-eye zone analysis, only stimuli located within DRIL or non-pathological areas were included to avoid confounding effects of coexisting biomarkers (ME or DROL). This grading was performed on a stimulus-by-stimulus rather than a patient level. It should be noted that these “zones” do not correspond to anatomical ETDRS subfields but represent modality-based classifications within DRIL eyes.

### Statistical analysis

Statistical analyses were conducted using IBM SPSS Statistics Version 27. Normality of the data was assessed using the Shapiro-Wilk test. A significance threshold of α = 0.05 was applied, and when necessary, Bonferroni corrections were used to adjust for multiple testing. Results are reported as mean values or estimated mean ± standard error (SE), as appropriate.

Intra-class correlation (ICC) coefficients were used to evaluate interreader reliability of the DRIL area measurements between the two graders. Additionally, inter-rater reliability of microperimetry grading was assessed using Cohen´s Kappa in a randomly selected subset of 10 patients.

For group comparison of RS, RS deviation and OCTA parameters between DRIL eyes and eyes without DRIL, linear mixed models with a random intercept per patient were applied. Adjusted models additionally included DRSS and diabetes duration as covariates.

For the intra-eye analysis of functional and structural parameters across predefined retinal regions within DRIL eyes (Zone I -III), analysis of covariance (ANCOVA) was performed with DRSS and diabetes duration as covariates, implemented as a mixed model with repeated measures to account for multiple stimuli per eye. The overall p-value refers to the main effect of retinal region within DRIL eyes, while pairwise comparisons (I vs. II, I vs. III, II vs. III) were Bonferroni-adjusted.

Pearson correlation analyses were used to evaluate associations between BCVA, RS, and OCTA parameters.

**Table 1:** Cohort characteristics.

	DRIL group	no DRIL group	*p*
Eyes, n (%)	27 (49.1)	28 (50.9)	
Age (years), mean ± SD	57.1 ± 14.6	58.2 ± 13.0	0.76
BCVA (logMAR), mean ± SD	0.4 ± 0.3	0.1 ± 0.1	**< 0.001**
Spherical equivalent, mean ± SD	+ 0.2 ± 0.9	- 0.1 ± 1.4	0.79
Type of DM			0.11
Type 1, n (%)	10 (37.0)	6 (21.4)	
Type 2, n (%)	17 (63.0)	22 (78.6)	
DRSS, mean ± SD	4.8 ± 0.5	1.2 ± 0.4	**< 0.001**
DRSS 1, n		22	
DRSS 2, n		6	
DRSS 3, n	1		
DRSS 4, n	3		
DRSS 5, n	23		
Current macular edema, n (%)	9 (33.3)	6 (21.4)	
Current or previous anti-VEGF therapy, n (%)	8 (29.3)	6 (21.4)	
Central subfield thickness, mean ± SD	294.4 ± 44.8	285.8 ± 33.8	0.43
Duration of DM (years), mean ± SD	19.7 ± 14.6	9.2 ± 7.0	**0.03**
HbA1c (%), mean ± SD	7.5 ± 0.8	7.0 ± 0.6	0.82
BMI (kg/m^2^), mean ± SD	28.9 ± 6.3	27.9 ± 5.2	0.71
DM therapy, n (%)			
Diet		5 (17.9)	
Oral medication	7 (25.9)	9 (32.1)	
Insulin	15 (55.6)	8 (28.6)	
Insulin + oral medication	5 (18.5)	6 (21.4)	
At least one additional cardiovascular risk factor, n (%)	23 (85.2)	21 (75.0)	0.22

Significant values are in [bold].

DM: diabetes mellitus; BCVA: Best-corrected visual acuity; DRSS: Diabetic Retinopathy Severity Scale; BMI: Body Mass Index.

**Table 2:** Functional and structural parameters of zones I, II, and III in DRIL patients.

	Adjusted mean ± SE in [dB]			Overall *p*-value	Difference between groups (*p*-value)		
	Zone I (*N*=100)	Zone II (*n*=46)	Zone III (*n*=1055)		Zone I vs. II	Zone I vs. III	Zone II vs. III
Microperimetry							
RS	13.07 ± 1.28	20.62 ± 0.79	23.11 ± 0.73	**< 0.001**	**0.016**	**0.002**	**0.02**
OCTA							
SCP: VD	6.31 ± 0.40	7.19 ± 0.21	9.24 ± 0.32	0.202	0.12	**< 0.001**	**< 0.001**
SCP: PD	18.45 ± 1.49	23.04 ± 0.97	32.59 ± 1.48	0.3	**0.003**	**< 0.001**	**< 0.001**
DCP: VD	7.21 ± 0.31	8.36 ± 0.28	9.6 ± 0.35	**0.036**	**0.05**	**< 0.001**	**< 0.001**
DCP: PD	21.16 ± 1.52	24.71 ± 1.39	33.04 ± 1.65	0.527	0.09	**< 0.001**	**< 0.001**

Values are adjusted means ± SE obtained from analysis of covariance (ANCOVA) with DRSS and time since diagnosis as covariates, using a mixed model with repeated measures. The p-value in the “overall p-value” column refers to the overall effect between zones. Pairwise comparisons (Zone I/II, I/III, II/III) were Bonferroni-adjusted. Sigmificance was set at *p* < 0.05 and shown in bold.

RS: Retinal sensitivity; SCP: Superficial capillary plexus; DCP: Deep capillary plexus; VD: Vessel density; PD: Perfusion density.

**Table 3:** Quantitative OCTA parameters in DRIL eyes and eyes without DRIL.

	DRIL group	No DRIL group	*p*
	Adjusted means ± SE		
SCP			
VD	7.36 ± 0.81	11.61 ± 0.63	**0.004**
PD	28.88 ± 3.2	42.92 ± 2.48	**0.02**
FAZ size	0.62 ± 0.21	0.33 ± 0.16	0.42
FAZ perimeter	3.72 ± 0.57	1.92 ± 0.44	0.08
FAZ circularity	0.56 ± 0.09	1.01 ± 0.07	**0.01**
DCP			
VD	6.93 ± 1.08	12.48 ± 0.08	**0.01**
PD	25.01 ± 5.26	44.15 ± 4.09	**0.04**
FAZ size	0.53 ± 0.27	0.58 ± 0.21	0.92
FAZ perimeter	3.08 ± 0.58	2.78 ± 0.45	0.76
FAZ circularity	0.55 ± 0.10	0.96 ± 0.08	**0.02**

Values are estimated means ± standard error obtained from the linear mixed model additionally including DRSS and duration of diabetes as covariates. Pairwise comparisons were Bonferroni-corrected. Significant results (*p* < 0.05) are shown in bold.

VD: Vessel density; PD: Perfusion density; FAZ: Foveal avascular zone; SCP: Superficial capillary plexus; DCP: Deep capillary plexus; DRIL: Disorganization of the retinal inner layers.

**Table 4:** Retinal sensitivity across retinal locations in DRIL eyes and eyes without DRIL.

	Adjusted means ± SE in [dB]	*p*
Retinal sensitivity		
DRIL area	17.6 ± 1.83	vs. no pathology: **< 0.001** vs. no DRIL: 0.27
No pathology in DRIL eye	27.6 ± 1.78	vs. no DRIL: 0.22
No DRIL	22.5 ± 1.13	
Retinal sensitivity deviation		
DRIL area	-8.9 ± 1.83	vs. no pathology: **< 0.001** vs. no DRIL: 0.16
No pathology in DRIL eye	+1.2 ± 1.78	vs. no DRIL: 0.31
No DRIL	-3.4 ± 1.12	

Results from linear mixed models with random intercept per patient adjusted for DRSS and diabetes duration as covariates. Reported *p*-values are from Bonferroni-corrected pairwise comparisons between retinal locations (DRIL area, non-pathological retina in DRIL eyes, and eyes without DRIL). Significance was set at *p* < 0.05 and shown in bold.

DRIL: Disorganization of the retinal inner layers.

## Supplementary Information

Below is the link to the electronic supplementary material.

Supplementary Material 1

## Data Availability

The data that support the findings of this study are available from the corresponding author upon reasonable request.
