# Supplementary material for: Increased axial resolution OCT improves structure-function correlation of the disorganization of the retinal inner layers in diabetic retinal disease
Source: Sci Rep. 2026 Jan 7;16:889. doi: 10.1038/s41598-025-34931-2 (PMC12783662; doi:10.1038/s41598-025-34931-2)

**Table S1**: **Unadjusted comparison of OCTA parameters between DRIL eyes and eyes without DRIL.**
Values are mean ± standard error. DRIL eyes showed significantly reduced VD, PD, and FAZ geometry in both SCP and DCP compared to eyes without DRIL (all p < 0.001). Significant values are shown in bold.

|  | DRIL group | no DRIL group | P |
| --- | --- | --- | --- |
| SCP |  |  |  |
| VD | 8.83 ± 0.22 | 10.62 ± 0.2 | **< 0.001** |
| PD | 33.95 ± 0.81 | 39.26 ± 0.75 | **< 0.001** |
| FAZ size | 0.64 ± 0.05 | 0.31 ± 0.04 | **< 0.001** |
| FAZ perimeter | 3.54 ± 0.13 | 2.09 ± 0.12 | **< 0.001** |
| FAZ circularity | 0.71 ± 0.02 | 0.89 ± 0.02 | **< 0.001** |
| DCP |  |  |  |
| VD | 8.8 ± 0.26 | 11.15 ± 0.24 | **< 0.001** |
| PD | 31.85 ± 1.22 | 38.91 ± 1.13 | **< 0.001** |
| FAZ size | 0.73 ± 0.06 | 0.43 ± 0.06 | **< 0.001** |
| FAZ perimeter | 3.47 ± 0.14 | 2.47 ± 0.13 | **< 0.001** |
| FAZ circularity | 0.69 ± 0.02 | 0.86 ± 0.02 | **< 0.001** |

Abbreviations: VD: Vessel density; PD: Perfusion density; FAZ: Foveal avascular zone; SCP: Superficial capillary plexus; DCP: Deep capillary plexus; DRIL: Disorganization of the retinal inner layers.

**Table S2**: **Unadjusted comparison of RS between DRIL regions, non-DRIL regions within DRIL eyes, and eyes without DRIL.**
Values are mean ± standard error. Retinal sensitivity and retinal sensitivity deviation were significantly lower in DRIL areas compared to non-DRIL areas within the same eye and to eyes without DRIL (all p < 0.001). Significant values are shown in bold.

| **Retinal sensitivity** | | adjusted means ± SE | **p** | |
| --- | --- | --- | --- | --- |
| DRIL area | 13.1 ± 0.57 | | vs. no pathology: **< 0.001**  vs. control: **< 0.001** |  |
| no pathology in DRIL eye | 23.1 ± 0.46 | | vs. control: **< 0.001** |  |
| no DRIL group | 25.3 ± 0.47 | |  |  |
| **Retinal sensitivity deviation** | | | |  |
| DRIL area | -13.2 ± 0.57 | | vs. no pathology: **< 0.001**  vs. control: **< 0.001** |  |
| no pathology in DRIL eye | -3.0 ± 0.46 | | vs. control: **< 0.001** |  |
| no DRIL group | -0.9 ± 0.47 | |  |  |

Abbreviations: DRIL: Disorganization of the retinal inner layers.

**Table S3**: **Correlation between BCVA and retinal parameters.**

Pearson correlation coefficients (r) and significance values (p) are presented for the relationships between BCVA (logMAR) and various retinal parameters. Parameters include retinal sensitivity [dB], VD and PD in the SCP and DCP as well as FAZ metrics such as size, perimeter, and circularity. Significance was set to p < 0.05, shown in bold.

|  | Pearson correlation | P |
| --- | --- | --- |
| **Microperimetry** | | |
| Retinal sensitivity [dB] | -0.50 | **<0.001** |
| **OCTA** | | |
| SCP: VD | -0.39 | **0.02** |
| SCP: PD | -0.39 | **0.02** |
| SCP: FAZ size | 0.40 | **0.01** |
| SCP: FAZ perimeter | 0.48 | **<0.001** |
| SCP: FAZ circularity | -0.39 | **0.02** |
| DCP: VD | -0.44 | **0.01** |
| DCP: PD | -0.40 | **0.01** |
| DCP: FAZ size | 0.29 | 0.09 |
| DCP: FAZ perimeter | 0.38 | **0.02** |
| DCP: FAZ circularity | -0.16 | 0.34 |

Abbreviations: VD: Vessel density; PD: Perfusion density; FAZ: Foveal avascular zone; SCP: Superficial capillary plexus; DCP: Deep capillary plexus; DRIL: Disorganization of the retinal inner layers

**Figure S1: Flowchart of patient inclusion for the DRIL and the no DRIL group.** A total of 48 patients with DRIL and 29 age-matched diabetic controls were initially screened. In the DRIL group, 21 patients were excluded due to insufficient image quality in OCT angiography and/or microperimetry, resulting in 27 patients eligible for final analysis. In the control group, one patient was excluded due to poor image quality, resulting in 28 patients included. The total study population consisted of 55 patients.


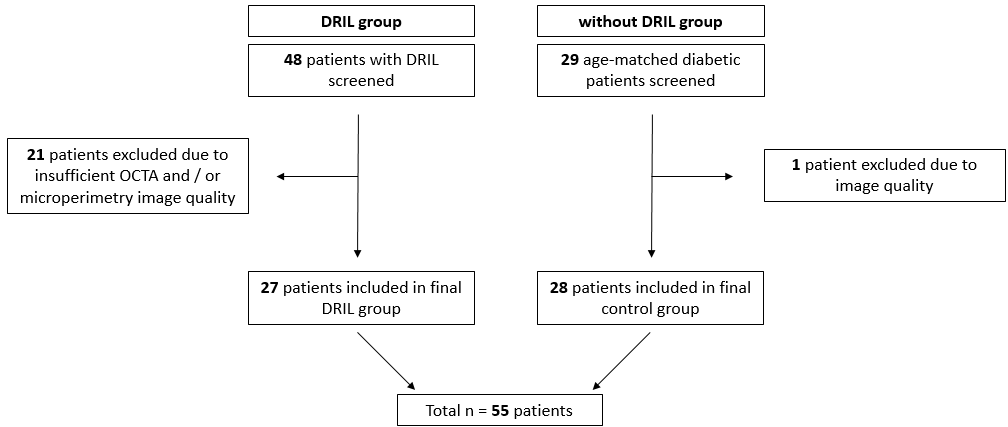


**Figure S2: Multimodal image alignment using Fiji.**

This figure illustrates the stepwise process used for aligning multimodal retinal images.

**Step 1:** Input images (OCTA image, near-infrared image with projected microperimetry stimuli, and en-face DRIL maps from High-Res and SD-OCT) were imported into FIJI. The *“*Register MP OCT” plugin (for microperimetry alignment) and the “Manual Landmark Selection” plugin (for OCTA alignment) were used. Initial alignment was performed by marking the same three vessel bifurcations in each image.
**Step 2:** Image dimensions were standardized to a common spatial scale (based on pixel dimensions and field of view), and alignment was refined using rigid transformation (translation and rotation only).
**Step 3:** Final registration accuracy was reviewed by two independent graders (K.W. and L.P.A.), and the alignment process was repeated if residual misalignments were detected.


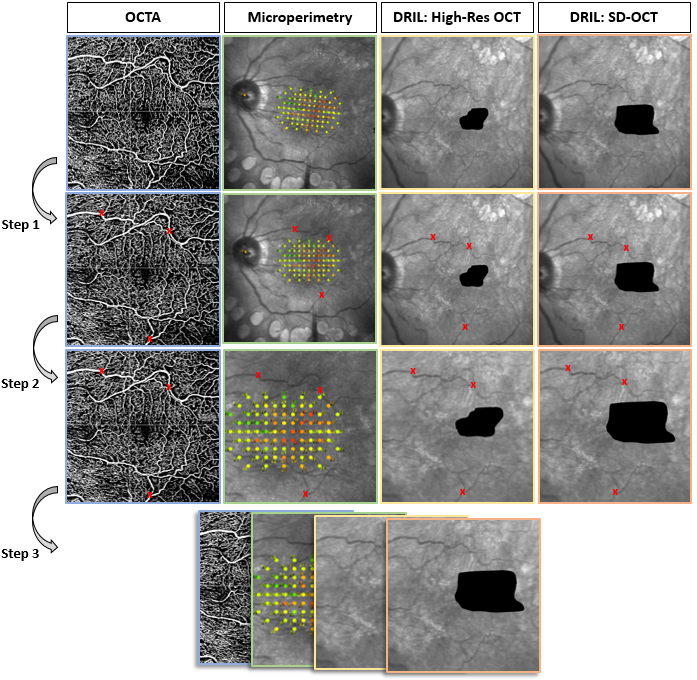

Supplement: Supplementary file 1 — Supplementary Material 1 [file 41598_2025_34931_MOESM1_ESM.docx]
